# Multislice CT angiography in cardiac imaging: prospective ECG-gating or retrospective ECG-gating?

**DOI:** 10.2349/biij.6.1.e4

**Published:** 2010-01-01

**Authors:** Z Sun

**Affiliations:** Discipline of Medical Imaging, Department of Imaging and Applied Physics, Curtin University of Technology, Perth, Western Australia

**Keywords:** Multislice computed tomography, coronary artery disease, radiation dose, diagnostic accuracy, electrocardiography-gating

## Abstract

With the advent of multislice CT more than a decade ago, multislice CT angiography has demonstrated a huge potential in the less invasive imaging of cardiovascular disease, especially in the diagnosis of coronary artery disease. The diagnostic accuracy of multislice CT angiography has been significantly augmented with the rapid technical developments ranging from the initial 4-slice, to the current 64-slice and 256 and 320-slice CT scanners. This is mainly demonstrated by the improved spatial and temporal resolution when compared to the earlier type of CT scanners. Traditionally, multislice CT angiography is acquired with retrospective ECG-gating with acquisition of volume data at the expense of increased radiation dose, since data is acquired at the entire cardiac cycle, although not all of them are used for postprocessing or reconstructions. Recently, there is an increasing trend of utilising prospective ECG-gating in cardiac imaging with latest multislice CT scanners (64 or more slices) with significant reduction of radiation dose when compared to retrospective ECG-gating method. However, there is some debate as to the diagnostic value of prospective ECG-gating in the diagnosis of coronary artery disease, despite its attractive ability to reduce radiation dose. This article will review the performance of retrospective ECG-gating in the diagnostic value of coronary artery disease, highlight the potential applications of prospective ECG-gating, and explore the future directions of multislice CT angiography in cardiac imaging.

## INTRODUCTION

Coronary artery disease (CAD) is the leading cause of death in western countries. The standard of reference for diagnosis of CAD is still invasive coronary angiography, with the advantage of high spatial resolution and temporal resolution. Despite its cost, inconvenience to patients, and a small but distinct procedure-related morbidity (1.5%) and mortality (0.2%) rate, more than 1 million invasive diagnostic coronary angiography procedures are performed annually in the United States alone. Similarly, CAD is the single most important cause of death in Australia and New Zealand. Every year, billions of dollars have been spent in the treatment of coronary artery disease ($3.9 billion direct spending in Australia 1993-94 according to Australian Institute of Health and Welfare, 2003). Each year cardiovascular disease resulted in 81,926 coronary angiography examinations in Australia 2001-02 (Australia’s Health 2004, AIHW). Given the invasiveness of coronary angiography and potential danger of having a small risk of serious complications (arrhythmia, stroke, coronary-artery dissection and death), a non-invasive technique for imaging of the coronary artery disease is highly desirable.

Imaging of the heart and coronary artery branches has always been technically challenging due to the heart’s continuous movement. Over the last decade, great strides have been made in the field of cardiac imaging as non-invasive coronary imaging modalities have undergone rapid developments [1-4]. Initially, electron-beam CT was found valuable in calcium scoring, but its application in the diagnosis of CAD was restricted to a greater extent due to limited spatial resolution [2]. Magnetic resonance imaging shows promising results as reported in some studies [3, 4], however, the imaging protocols are variable based on the MR vendor and software availability which prevents it from being widely used. Imaging of the heart has moved into a new diagnostic era with the introduction of multislice CT (MSCT) and development of electrocardiography-synchronised scanning and reconstruction techniques.

Multislice CT represents technical evolution in cardiac imaging when 4-slice CT scanner was first introduced into the clinical practice in 1998 [5]. Diagnostic accuracy of multislice CT in CAD has been significantly improved with the development of scanning techniques, which are demonstrated by the emergence of 16-, 64-slice and even more recently 256-and 320-slice CT scanners [6-9]. Until recently, all of the studies were performed with retrospective ECG-gated cardiac imaging with high diagnostic accuracy for the detection of CAD at the cost of high radiation dose since images were acquired during the entire cardiac cycle. Prospective gating with axial non-helical scan was used a long time ago with electron-beam CT for calcium scoring. However, in early MSCT of the coronary arteries, the retrospective approach was favoured (and is still widely regarded) as the method of choice to achieve high image quality, especially when patient factors are not favourable for optimal image quality (e.g. arrythmias, high heart rate, etc). The main drawback of the retrospective approach is the relatively higher dose penalty and this has brought back into favour the prospective approach.

Recently, prospective ECG-gating was introduced for cardiac MSCT angiography, and this imaging protocol is increasingly being reported in the literature, despite sufficient evidence still needed to verify its diagnostic value. This paper will review the diagnostic accuracy of retrospective gating CT angiography; the potential applications of prospective gating cardiac CT angiography, and highlight some future directions of MSCT in cardiac imaging.

## DIAGNOSTIC VALUE OF RETROSPECTIVE ECG-GATED CT ANGIOGRAPHY IN CAD

The feasibility of cardiac MSCT was initially demonstrated with 4-slice CT using retrospective ECG-gated technique. Volumetric CT data is acquired throughout the entire cardiac cycle during simultaneous recording of the ECG signal. Subsequently, data from specific periods of the cardiac cycle (most commonly at late diastolic phase) is reconstructed by retrospective referencing to the ECG signal with the aim of generating images with the least motion artefacts. Over the last decade a great deal of interest has been focused on imaging and diagnosis of CAD with MSCT due to its less invasive nature and fast scanning technique with extended z-axis coverage when compared to single slice CT. Earlier studies with 4-slice CT showed moderate diagnostic accuracy with pooled sensitivity and specificity of 78% and 93%, respectively [10]. However, image quality was impaired in many cases with 4-slice CT due to limited spatial and temporal resolution, and the unassessable segments could be as high as more than 20% in 4-slice studies [10]. With the introduction of 16-slice CT, image quality in coronary MSCT has become more consistent with improved results achieved. Studies that used 16-slice CT with acquisition and rotation times of <400 ms have reported sensitivities between 83% and 98% and specificities between 96% and 98% [11-14].

Shorter examination times are possible with further improved diagnostic accuracy with 64-slice CT owing to improved spatial and temporal resolution compared with 16-slice CT. Acquisition of isotropic volume data are made available with 64-slice CT, thus detection of main and side coronary artery branches is improved when compared to earlier types of MSCT scanners. Several meta-analyses of 64-slice CT studies reported sensitivities of 93% and specificities of 96% (in 6 studies) [15], sensitivities of 97% and specificities of 88% (in 15 studies) [16], and sensitivities of 86% and specificities of 96% (in 19 studies) [17]. These studies concluded that MSCT, especially with 64-or more slice CT, has high diagnostic accuracy for detection of CAD and could be used as an effective alternative to invasive coronary angiography in selected patients.

In 2006, the first dual source multislice CT scanner was introduced [18]. With the coupling of two X-ray tubes mounted at 90° to each other in a single gantry, the rotation time was shortened, temporal resolution was doubled (83 ms with dual source CT vs 165 ms with 64-slice CT), and heart rate dependence was eliminated. Studies performed with dual source CT showed promising results with high diagnostic accuracy for detection of CAD, and most importantly the image quality is independent of heart rate [19-21]. Leber *et al.* [19] in their early study reported dual source CT had high diagnostic accuracy for detection of coronary stenoses and image quality was independent of heart rate. The benefit of improved temporal resolution with dual source CT is evident with supporting evidence by later studies further confirming its improved accuracy, with heart rate having no significant effect on image quality and diagnostic accuracy [19-22]. Rixe *et al.* in their recent study concluded that high diagnostic accuracy (99% and 92% at segment-based and patient-based analysis, respectively) was achieved even at high heart rates [22]. Heart-rate independent image quality with dual source CT represents another milestone in cardiac CT and it could be used as a reliable alternative to invasive coronary angiography for detection of CAD.

While satisfactory results have been achieved with the retrospective ECG-gating through continuous exposure in a low-helical pitch (0.2-0.4) resulting in multiple overlapping regions of X-ray exposure, the downside is the relatively high effective radiation doses. The radiation dose associated with retrospective ECG-gating is gradually increased with the increased number of detector rows and reduction of detector size. Thus, 4-slice CT scanners have lower dose than 16-slice scanners. Similarly 16-slice scanners have lower doses than 64-slice, and subsequently doses from 64-slice will be lower than those acquired with 256 and 320 slice scanners. Therefore, various strategies have been taken to reduce the radiation dose while using MSCT angiography in cardiac imaging, and prospective ECG-gating is by far the most effective and significant technique to reduce radiation dose.

## DIAGNOSTIC APPLICATION OF PROSPECTIVE ECG-GATING CT ANGIOGRAPHY IN CAD

Prospective ECG-gating utilises the same technique as that used in electron-beam CT which is defined as the step and shoot method. It is mainly used for quantification of calcium burden, but recently it is increasingly used for CT coronary angiography examinations. The scan is performed in a non-helical way with acquisition of a series of axial images instead of volumetric data. Unlike retrospective gating, prospective ECG-gating or prospective triggering allows for acquisition of data by selectively turning the x-ray tube on only in the selected phase, triggered by the ECG signal, and turning off during the rest of R-R cycle. This is also referred to as sequential or step-and-shoot acquisition with prospective triggering and the effective pitch is 1.0. The main advantage of this scanning protocol is the lower radiation dose as X-ray exposure only takes place during the selected cardiac phase rather than throughout the entire cardiac cycle. Therefore, a significant reduction of radiation dose can be expected from prospective ECG-gating, which is the most attractive side of this scanning protocol compared to retrospective ECG-gating.

Recent technical developments of MSCT imaging technique allows for prospective ECG-gating to be performed in a single heart beat with helical scan [9, 23, 24]. Rybicki in their initial study showed that using 320-slice CT (Toshiba AquilionOne Dynamic Volume CT) prospective ECG-gating diagnostic images were achieved in more than 90% of patients with reduction of radiation dose [9]. In addition to reduction of motion artefacts, functional assessment of the heart can also be achieved with 320-slice CT since the scan is performed in a single heart beat [24].

Use of prospective ECG-gating with 64-slice or dual source CT has been reported to reduce the effective radiation dose by up to 90% when compared to retrospective ECG-gating technique [25-37]. In 2006, Hsieh *et al.* [25] first described a step-and-shoot prospectively gated protocol for imaging coronary artery disease. They claimed that patient dose could be reduced by at least 50% when compared to the standard retrospective gated protocol without compromising image quality. Afterwards, Husmann *et al.* [24] in their first clinical experience demonstrated the feasibility of prospective ECG-gating with low dose results. Diagnostic image quality was achieved in 93% of 41 patients with suspected or proved CAD with very low mean effective dose of 2.1 mSv (1.1-3.0 mSv), when heart rate was less than 63 bpm. This contrasts significantly to the higher radiation dose arising from previous retrospectively gated cardiac MSCT angiography (up to 21 mSv) [27, 28]. It must be noted that such a high radiation dose results from scanning without ECG-based tube current modulation. The mean effective doses were reduced to less than 10 mSv for 64-slice CT coronary angiography performed with ECG-based tube current modulation [38, 39], although it is still much higher than those from prospective gating protocols.

Studies comparing prospective and retrospective gating further confirmed the significant reduction of radiation dose resulting from the former imaging protocol. Shuman *et al.* in their prospective study compared a group of patients who underwent prospectively gated cardiac CT with another matched group of patients who underwent retrospectively gated cardiac CT. Similar image quality of coronary segments was scored for both groups, but 77% radiation dose reduction was achieved in the prospectively gated group [29]. This was also confirmed by another two comparative studies performed by Hirai *et al.* and Earls *et al.* who both showed the similar image quality between prospective and retrospective gated imaging, but with dose reduction of 79% and 83% achieved in the prospective groups [31, 32]. Most recently, Earls *et al.* reported their experience of using prospective gating in the largest clinical group including more than 2000 cases [32]. With adequate preparation and patient selection, they concluded that most patients would benefit from prospective gating with acceptable diagnostic images and significant reduction of effective radiation dose, subsequently reducing the risk of developing radiation-induced malignancies.

Radiation dose can be further reduced with reduction of the tube voltage (kVp), in addition to the tube current modulation which is available in most of the 64-slice scanners. Researchers investigated the prospective gating with different kVp, and its effect on radiation dose, and results are promising. Stolamann *et al.* studied the image quality and radiation dose with dual source CT prospective gating by using different protocols [36]. Their results showed no significant differences in image quality between 100 kV and 120 kV protocols, but with significant reduction of radiation dose achieved in 100 kV protocols (1.2 mSv ± 0.2) compared with 120 kV protocols (2.6 mSv ± 0.5). Gopal *et al.* in their recent study consisting of 149 patients compared prospective gating and retrospective gating protocols with different kV groups [34]. Their results showed that a reduction of radiation exposure up to 90% was achieved with use of 100 kVp and when compared to the conventional prospective gating at 120 kVp. Therefore, there is still room for radiation dose reduction when using the prospective gating technique, although more data from multicentres are needed to corroborate these early findings.

It is important to note that while prospective gating leads to a significant reduction in effective radiation dose and provides equivalent or improved image quality relative to retrospective gated images, studies reported in the literature highlight some important limitations to the current 64-slice CT (including dual source). The main limitation lies in the fact that image quality is dependent on the heart rate, heart rate variation and body mass index (BMI). Maximum heart rate threshold is between 63-75 bmp for prospective gated imaging. When heart rate is greater than 70 bmp, heart rate variation greater than 10 bmp, or BMI greater than 30 kg/m^2^, lower image quality occurs as reported by recent studies [35, 36]. All of these limitations indicate that prospective gating is limited to patient cohorts strictly defined by the above three factors, thus the prospective gating protocol applies only to appropriately selected patients.

Further technical developments in MSCT technique overcome the abovementioned limitations with the emergence of new generation of MSCT techniques such as 256- and 320-slice CT scanners. Longer z-axis coverage available with 256 and 320-slice scanners ranging from 12.8 cm to 16 cm in one gantry rotation permits full cardiac coverage in one gantry rotation with prospective gating, thus, eliminating the restrictions and limitations associated with 64-slice scanners [9, 39-41]. Weigold *et al.* reported the superiority of 256 prospective gating CT in their initial experience with high image quality, low dose, independent of higher heart rates and higher BMI [41]. Studies by others using 320-slice CT further demonstrated the improvement of prospective gating with the new generation of CT scanners [23, 24]. The majority of patients could be imaged in a single heartbeat with excellent image quality, according to the study performed by Rybicki *et al.* [9]. Also, patients with cardiac arrhythmias are no longer excluded from the cardiac CT imaging [24].

## DIAGNOSTIC ACCURACY OF PROSPECTIVE ECG-GATED CT ANGIOGRAPHY IN CAD

Despite the promising aspect of significant reduction of radiation dose with prospective gating, there is lack of sufficient evidence to show the diagnostic value or performance of prospective gating in the detection of coronary artery disease. Most of the studies currently available in the literature addressed the image quality and reduction of radiation dose when comparing prospective with retrospective gated protocols. A direct comparison between prospective gating and conventional coronary angiography is scarce and data is limited for the diagnosis of coronary artery stenosis. Scheffel *et al.* presented the first report demonstrating the diagnostic performance of low-dose prospective gating CT for the diagnosis of CAD [37]. Diagnostic accuracy was obtained in patients with heart rate less than 70 bmp with prospective gated coronary MSCT with more than 96% sensitivity and specificity, whether the analysis was segment-based, vessel-based or patient-based. Stolzmann *et al.* also reported the high diagnostic accuracy of prospective gating for diagnosis of CAD with low radiation dose, even in the presence of heavy calcification, despite the fact that increased rate of non-diagnostic segments was observed due to heavy calcification [42]. Further studies comparing prospective gating with gold standard coronary angiography are required to verify the diagnostic value of this rapidly growing CT imaging protocol.

Apparently, there are two important limitations of prospective gating performed with 64-slice CT: prospective gating is limited to heart rates lower than 75 bpm due to short z-axis coverage. The z-axis coverage during image acquisition with 64-slice CT is only 4 cm, thus three to five dataset are required subsequently to image the entire heart. Stair-step artefacts due to misalignment of two adjacent datasets may happen if the position of the patient on the CT table changes during table travel, or heart rate is irregular or heart rate variation is great during the scan. Husmann *et al.* noticed that 62% of patients have stair-step artefacts in prospective gated coronary CT angiography of coronary arteries [43]. The stair-step artefacts are mainly determined by the motion of the patient during the CT scan and by motion of the heart, especially since heart rates are apparently variable. Second, cardiac images are acquired during only a small portion of the R-R interval; thus, functional information about cardiac valve motion or wall motion is not available.

The first limitation has been addressed with the use of 256 or 320 slice CT as shown by some studies mentioned above [8, 9, 23, 24]. With extended z-axis coverage, images can be acquired within a single gantry rotation, thus eliminating the table movement during data acquisition, consequently eliminating the stair-step artefacts. The limitation of no functional information has also been overcome with the use of the new generation of CT technique since myocardial perfusion imaging can be obtained with 256 or 320-slice prospective gating. Kitagawa *et al.* in their initial experience demonstrated that 320-slice CT allows for simultaneous perfusion imaging for the entire myocardium [24]. Early studies showed the accuracy of 256- and 320-slice CT perfusion imaging for the simultaneous evaluation of coronary atherosclerosis and its physiological significance with a mean dose of 13.5 ± 3.5 mSv [44, 45]. Apparently the radiation dose is higher than that acquired with 64-slice prospective gating technique, thus, further technical improvement to reduce radiation dose is necessary.

## RETROSPECTIVE OR PROSPECTIVE GATED IMAGING FOR MSCT IN CAD?

It seems that the current direction of using MSCT in cardiac imaging is moving from previous retrospective gating to prospective gating, and this is demonstrated by the increasing reports available in the literature over the last few years. The main driving force for this trend is the reduction of radiation dose, which is the most attractive aspect of prospective gating technique. Certainly this is only made available due to the technical developments of multislice CT technique, especially with increased temporal resolution. In order to acquire diagnostic quality images, prospective gating CT scans must be performed with use of 64- or more slice CT scanners. While radiation dose is significantly lower than that acquired with retrospective gating technique, the evidence of prospective gating for diagnosis of coronary artery disease is scarce at this stage. Thus, it is too early to draw conclusions that prospective gating can be used as a reliable alternative to conventional coronary angiography for the diagnosis of coronary artery disease before adequate research evidence is available.

With more research findings available in the literature in the near future, it is expected that multislice CT will be used as a first line technique in cardiac imaging, possibly with prospective gating replacing the traditional retrospective gating technique. There is no doubt that multislice CT has entered a new era in cardiac imaging with the advent of 64-, 256- and 320-slice scanners, and its applications in clinical practice will benefit more patients suspected of coronary artery disease. While reduction of radiation dose is important, the most important aspect of cardiac CT is the image quality required for diagnostic purposes. Thus, both of these two factors need to be taken into account when choosing prospective gating CT coronary angiography in the diagnosis of coronary artery disease (Table 1). The following recommendations aim to provide some kind of guidance for readers using multislice CT angiography in cardiac imaging:

**Table 1:** Comparison of prospective ECG-gating and retrospective ECG-gating for diagnosis of coronary artery disease (with 64- or more detector row scanners).

Parameters to be compared	Retrospective ECG-gating		Prospective ECG-gating	
	Pros	Cons	Pros	Cons
Scanning protocols	Axial helical scan allows acquisition of volume data	Exposure takes place during the entire cardiac cycle and only a portion of data is used for reconstruction	Exposure only occurs at a selected cardiac cycle (late diastolic phase)	Axial non-helical scan with most of the manufacturers; thus no volume data is available
Image quality (assessable segments)	98-100%	Affected by heavy calcification and high heart rate	95-99%	Affected when heart rate is >70 bpm
Effect of heart rate	Diagnostic accuracy is high even in higher heart rate; Independent of heart rate with dual source CT	Diagnostic accuracy slightly decreases with increasing heart rate (70-100 bpm)	High assessable segments and diagnostic value in low heart rate	Limited to heart rate <70 bmp; Limited to regular and stable heart rate
Diagnostic value	High sensitivity and specificity, especially very high negative predictive value	Sensitivity is affected by heavy calcification	High diagnostic accuracy, although the data is scarce at the moment	Very limited data available
Radiation dose	Online tube current modulation could reduce radiation dose	High radiation dose with range of 7.6-31.8 mSv	Significant reduction of with range of 2.1-9.2 mSv	
Cardiac functional assessment	Available as volume data are acquired		Functional assessment is only available with 256- or 320-slice CT	Unavailable with 64-slice CT scanners

- With use of 64-slice or dual source CT, in patients suspected of CAD for whom only the cardiac anatomy or presence or absence of CAD is the main concern, prospective gating is suggested;
- With use of 64-slice or dual source CT in patients suspected of CAD, if cardiac functional information will make a meaningful or significant contribution to the CT assessment or clinical treatment, use of retrospective gating is suggested with additional dose being justified;
- With use of 256-or 320-slice CT in cardiac imaging, prospective gating is recommended since it allows acquisition of dataset in one gantry rotation, thus providing both anatomical assessment and physiological evaluation;
- With use of prospective gated protocol, 100 kVp is recommended in patients with BMI less than 30 for further reduction of the effective radiation dose;
- Narrowing the phase window width in prospective gated protocol is recommended to reduce patient radiation dose in a single heartbeat CT angiography (256- or 320-slice CT).
